# Effects of cervical mobilization and exercise on pain, movement and function in subjects with temporomandibular disorders: a single group pre-post test

**DOI:** 10.1590/1678-775720150240

**Published:** 2016

**Authors:** Letícia Bojikian CALIXTRE, Bruno Leonardo da Silva GRÜNINGER, Melina Nevoeiro HAIK, Francisco ALBURQUERQUE-SENDÍN, Ana Beatriz OLIVEIRA

**Affiliations:** 1 - Universidade Federal de São Carlos, Departamento de Fisioterapia, Laboratório de Cinesiologia Clínica e Ocupacional, São Carlos, SP, Brasil.; 2 - Universidade Federal de São Carlos, Departamento de Fisioterapia, Laboratório de Avaliação e Intervenção do Complexo do Ombro, São Carlos, SP, Brasil.; 3 - Universidad de Salamanca, Departamento de Enfermería y Fisioterapia; Instituto de Investigación Biomédica de Salamanca, Salamanca,España.

**Keywords:** Temporomandibular joint, Neck, Physical therapy specialty, Stomatognathic system

## Abstract

**Objective:**

To investigate the effect of a rehabilitation program based on cervical mobilization and exercise on clinical signs and mandibular function in subjects with temporomandibular disorder (TMD). Material and Methods: Single-group pre-post test, with baseline comparison.

**Subjects:**

Twelve women (22.08±2.23 years) with myofascial pain and mixed TMD according to the Research Diagnostic Criteria for Temporomandibular Disorders.

**Outcome measures:**

Subjects were evaluated three times: twice before (baseline phase) and once after intervention. Self-reported pain, jaw function [according to the Mandibular Functional Impairment Questionnaire (MFIQ)], pain-free maximum mouth opening (MMO), and pressure pain thresholds (PPTs) of both masseter and temporalis muscles were obtained. Baseline and post-intervention differences were investigated, and effect size was estimated through Cohen’s d coefficient.

**Results:**

Jaw function improved 7 points on the scale after the intervention (P=0.019), and self-reported pain was significantly reduced (P=0.009). Pain-free MMO varied from 32.3±8.8 mm to 38±8.8 mm and showed significant improvement (P=0.017) with moderate effect size when compared to the baseline phase. PPT also increased with moderate effect size, and subjects had the baseline values changed from 1.23±0.2 kg/cm^2^ to 1.4±0.2 kg/cm^2^ in the left masseter (P=0.03), from 1.31±0.28 kg/cm^2^ to 1.51±0.2 kg/cm^2^ in the right masseter (P>0.05), from 1.32±0.2 kg/cm^2^ to 1.46±0.2 kg/cm^2^ in the left temporalis (P=0.047), and from 1.4±0.2 kg/cm^2^ to 1.67±0.3 kg/cm^2^ in the right temporalis (P=0.06).

**Conclusions:**

The protocol caused significant changes in pain-free MMO, self-reported pain, and functionality of the stomatognathic system in subjects with myofascial TMD, regardless of joint involvement. Even though these differences are statistically significant, their clinical relevance is still questionable.

## INTRODUCTION

Temporomandibular disorders (TMD) are defined by the American Academy of Orofacial Pain as a collective term for a number of clinical problems involving the masticatory musculature, the temporomandibular joints (TMJs), and their associated structures. This dysfunction impairs chewing, swallowing, and speaking, and the main signs are joint noises, reduced range of motion, and mandibular deviation during TMJ function.

The relationship between the TMJ and the cervical spine can be explained by the neuroanatomical convergence of nociceptive neurons that receive trigeminal and neck sensory inputs^24^. In primates, this is caused by the topographic arrangement of the trigeminal caudate nucleus that allows information exchange between the spinal and trigeminal nerves. Therefore, stimulation of structures innervated by the trigeminal nerve may produce neck pain and vice-versa^6^.

The association between neck pain (NP) and TMD has been widely investigated^2^. A strong relationship was demonstrated between neck disability and jaw dysfunction in patients with TMD with altered electromyographic activity of the esternocleidomastoid and anterior scalene muscles during the craniocervical flexion test when compared with healthy controls^3^. Moreover, TMD patients presented reduced endurance of neck flexors and extensor muscles. Bevilaqua-Grossi, et al.^7^ (2007) suggested that signs and symptoms of NP can perpetuate TMD, but they do not appear to predispose the subject to the dysfunction. There seems to be a positive, yet still controversial, association between NP and TMD in adults. The elucidation of this cause and effect association will reinforce the possibility for physical therapists to assist patients with TMD by approaching their cervical spines^25^ ^,^ ^29^.

Physiotherapy techniques involving manual therapy, active and passive stretching, strengthening of involved muscles, and postural exercises seem to be effective for TMD treatment^28^. According to a systematic review, manual therapy has been applied directly on TMJ structures, indirectly on the cervical or thoracic spine, or on both regions or structures when composing manual therapy protocols^9^. Therapeutic approaches using manual therapy and exercises on the cervical spine have shown benefits for pain, maximum mouth opening (MMO), and pressure pain thresholds (PPTs)^24^. However, studies examining physical therapy interventions are still required to strengthen the evidence of their effect on complementing TMD treatment^28^.

The highest methodological quality studies provide evidence supporting the use of high-velocity and low-amplitude thrust manipulations on the upper cervical spine to improve PPT and MMO on TMD patients^27^ ^,^ ^29^. However, such evidence is not available when considering nonmanipulative and exercise techniques. La Touche, et al.^24^ (2009) applied manual therapy and exercise to the cervical spines of patients with myofascial TMD and reported improvement of clinical signs and symptoms, although the lack of a control group, placebo, or baseline phase compromises the evidence of their results. It is also unclear how subjects with mixed TMD (combining myofascial with joint involvement) would respond to this treatment.

Therefore, the aim of this study was to investigate the effects of a protocol based on cervical mobilization and exercise on mandibular function, PPTs, self-reported pain and MMO in subjects with myogenic or mixed TMD compared to a wait-and-see period (baseline). The hypothesis of the study is that these outcomes will improve after intervention when compared to the baseline phase.

## METHODOLOGY

### Study design

This single-group pre-post test was conducted over a 9-week period. The baseline phase consisted of two evaluations (E1 and E2) performed with a 3-week interval, during which time the subjects received no treatment. The intervention phase consisted of 10 sessions of physical therapy over a 5-week period (the first session occurring up to 7 days after E2), and a third evaluation (E3) was performed 3–5 days after the last session.

Although methodological limitations are inherent to single-group pre-post test studies (i.e., the inability to control threats to internal validity), the use of a baseline phase was proposed to strengthen the study design. Consequently, the stability of the outcome measures was assessed and allowed subjects to act as their own controls^5^.

### Subjects

Participants were eligible to participate if they were older than 18 years of age and had a primary diagnosis of myofascial pain with or without limitation of mouth opening according to Axis I of the Research Diagnostic Criteria for TMD (RDC/TMD). They could also be eligible when there was unilateral or bilateral joint impairment or disc displacement associated to miofascial pain. These criteria demonstrated good reliability (ICC 0.51- 0.60), especially on myofascial pain diagnoses^20^. Subjects were excluded if they presented with any of the following: a diagnosis of isolated disc displacement, arthrosis, or arthritis of the TMJ according to the RDC/TMD without associated myofascial commitment; a history of mandibular or neck traumatic injuries; fibromyalgia syndrome; a diagnosis of systemic disease (rheumatoid arthritis, systemic lupus erythematosus, or psoriatic arthritis); the presence of neurological disorders; and a history of any form of treatment (physiotherapy, splint therapy, or acupuncture) within the 3 months before the study. They were asked not to use pain medication or muscle relaxants at least 24 hours before the evaluations and during the treatment period.

The severity of the signs and symptoms of TMD was investigated in a previous study^9^ among all physiotherapy undergraduate students at the University (n=116) through the Fonseca Anamnestic Index. This is a simple, fast, and low-cost alternative to screen subjects displaying signs and symptoms of the disorder, as well as to classify the severity of the symptoms (absent, light, moderate, or severe). It is validated to Brazilian Portuguese language and has a regular reliability (ICC 0.55)^10^.

Twenty-three students showed moderate (n=15) or severe (n=8) signs and symptoms of the disorder, according to the Fonseca Anamnestic Index, and they were invited to participate in the diagnostic process. The evaluation proposed by RDC/TMD was performed by a physiotherapist who had recently graduated and was trained and supervised by two physiotherapists with at least 10 years of experience in physical therapy treatment and evaluation. Eleven subjects were excluded because of the absence of myofascial pain (n=2); a lack of a TMD diagnosis (n=2); and a lack of interest in participating in the intervention (n=7).

**Figure 1:**
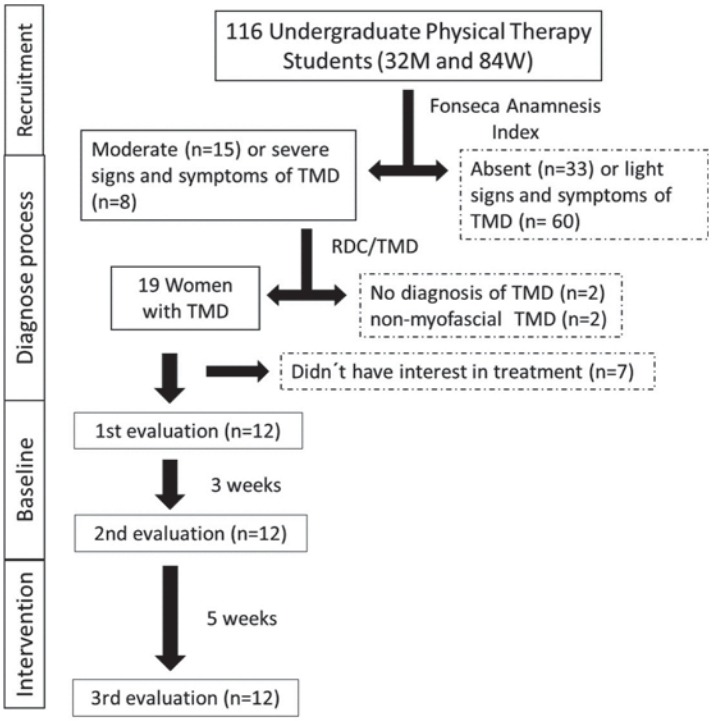
Flowchart showing the phases of the study and number of subjects. The dotted frames indicate the excluded subjects

The final sample was composed of 12 women with a mean age of 22.08±2.23 years (Figure 1). They were diagnosed with myofascial pain with limitation of mouth opening (n=2), or mixed TMD (n=10) - combining myofascial pain either with joint impairment (n=7) or disc displacement (n=3). All subjects had bilateral myofascial involvement, although most joint or disc disorders were unilateral (six left sided, two right sided).

All research procedures were approved by the local Ethics Committee on Human Research (Authorization #145/2012). The study was registered on ClinicalTrials.gov (NCT01954511).

### Evaluation protocol

#### Evaluation of mandibular function

Mandibular function was evaluated with the *Mandibular Functional Impairment Questionnaire* (MFIQ)*.* This questionnaire has 17 questions, each one scoring between 0 and 4. The higher the score, the greater the functional impairment. The sum of the responses was used in the statistical analysis. The Portuguese version used in this study has shown good reliability^11^ it was applied to 62 individuals who completed the questionnaire on two occasions. Validity and reliability of the data gathered with MFIQ were evaluated in a sample of 249 patients. Construct-related validity was assessed through factorial validity (by means of a confirmatory factor analysis).

#### Evaluation of clinical signs of TMD

Clinical signs of TMD were evaluated according to RDC/TMD (http://www.rdc-tmdinternational.org). Pain-free MMO was measured with a 0.05 mm precision analog vernier caliper. Participants were seated while the assessor asked them to open their mouths as much as possible without causing pain. At the limit of pain-free mouth opening, the distance between the upper-lower central incisors (not considering the overbite) was measured.

Self-reported pain was evaluated through the numeric scale presented in RDC/TMD. Subjects were asked to report their pain at the moment on a scale ranging from 0 (no pain) to 10 (worst pain ever experienced), with a 1-point interval.

#### Evaluation of PPTs

Masseter and anterior temporalis PPTs were bilaterally assessed using an analog algometer (Pain Diagnosis and Treatment Inc., Great Neck, NY, USA). The measurements were reported in kg/cm^2^. According to literature, the reliability of this method is high [ICC=0.91 (95% confidence interval: 0.82-0.97)] for healthy subjects^13^ at a rate of 5 Newtons, and moderate (ICC=0.64) for TMD patients^17^. The points were always evaluated in the same order and repeated three times, with a 1-minute interval. The average of the three measurements was considered for each point.

The masseter muscle was evaluated at 1 cm above and 2 cm anterior to the mandibular angle. The anterior temporalis muscle was evaluated at 2 cm above the zygomatic arch, between the lateral edge of the eye and the anterior part of the fibers^24^.

#### Intervention

The protocol reported by La Touche, et al.^24^ (2009) was used as a reference. Therefore, muscle-conditioning techniques, manual therapy, and stretching were applied for 10 sessions of approximately 35 minutes each: 20 minutes of manual therapy, 10 minutes of muscle conditioning exercises, and 5 minutes of muscle stretching.

**Figure 2:**
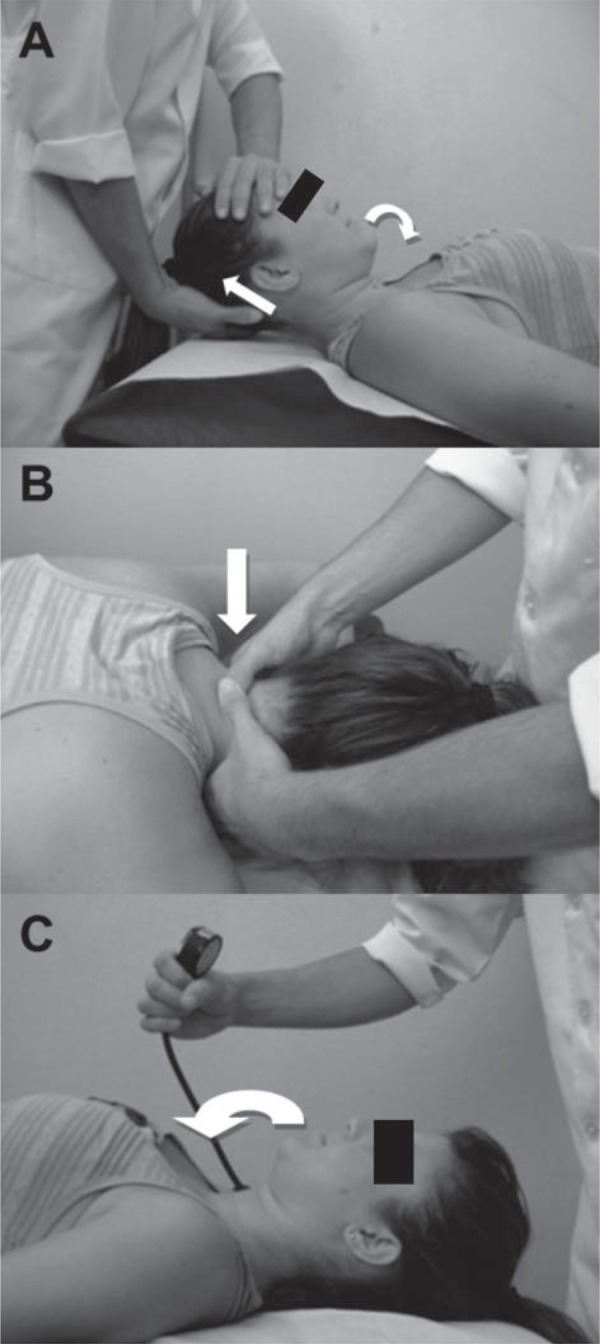
Positioning of patient and therapist during (A) upper cervical flexion mobilization, (B) C5 central posterior-anterior mobilization, and (C) craniocervical flexor stabilization exercise. The arrows indicate the direction of movement

The techniques were applied in the following order:

Upper cervical flexion mobilization: the subject lay down in the supine position (Figure 2A) while the therapist kept one hand in contact with the occipital bone, exerting traction force, and placed the other on the frontal region of the subject’s head, applying caudal pressure. The combined forces promoted flexion on the upper cervical region with the mobilization being applied at a slow rate of 2 seconds *per* oscillation for a total time of 10 min^24^.

C5 central posterior-anterior mobilization: the subject lay down in the prone position, with neutral cervical spine position (Figure 2B). The therapist placed the tips of his thumbs on the posterior surface of the C5 spinous process while the other fingers were gently rested around the subject’s neck. The oscillations were conducted in the third grade of the Maitland scale at the frequency of 2 oscillations *per* second and performed for 9 minutes, divided into 3 sets of 3 minutes, with a 1-minute interval.

Craniocervical flexor stabilization exercise: lying down in the supine position, the subject was instructed to perform craniocervical flexion (Figure 2C). The head was kept in contact with the supporting surface to facilitate activation of deep neck flexors, with minimal activity of the superficial neck flexor muscles^21^. This contraction was monitored using a pressure sensor (Stabilizer; Chattanooga Group, Inc., Chattanooga, TN, USA). The therapist monitored any contraction of superficial neck flexors muscles with palpation of the anterior neck region to ensure that the exercise was being correctly performed. Each craniocervical flexion produced a pressure ranging from 20 to 22 mmHg. The subjects were instructed to maintain that pressure using visual feedback for 10 seconds with no contraction of superficial neck flexor muscles. This procedure was repeated 10 times. Load increase was used to progress the exercise. The number of repetitions and duration of each contraction was constant^21^.

**Figure 3:**
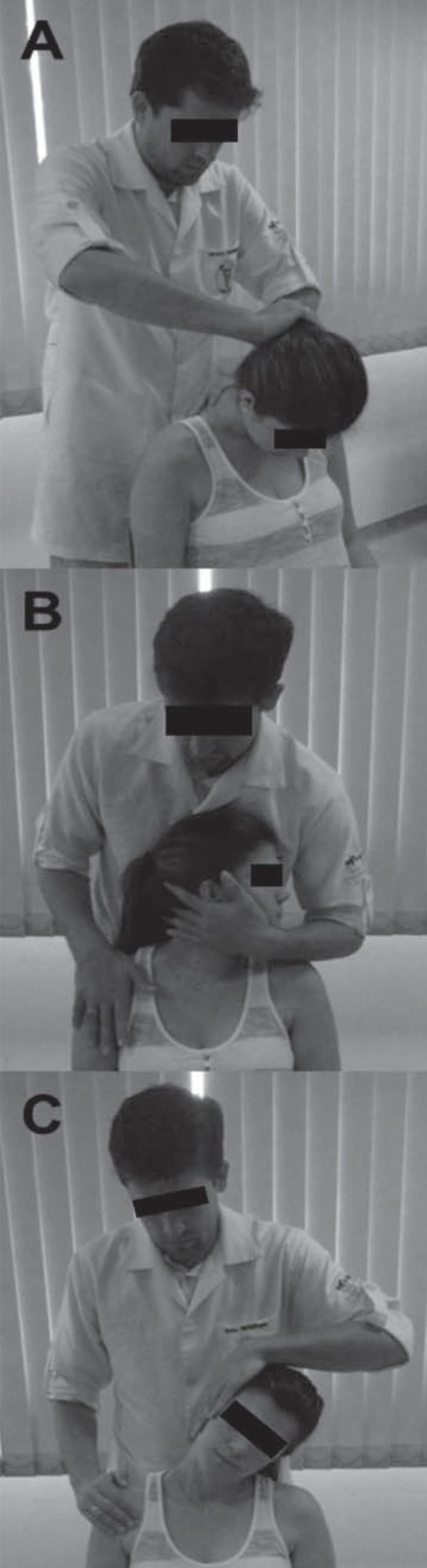
Positioning of the patient and the therapist during stretching exercises: (A) semispinalis capitis and splenius capitis stretching; (B) sternocleidomastoid muscles and scalenes stretching, and (C) upper trapezius stretching

Stretching exercises: while seated, the subject performed stretching exercises for the upper trapezius, scalene, semispinal muscle of head, splenius capitis, and sternocleidomastoid muscles (Figure 3). These muscles are directly involved with head positioning and their shortening produces misalignment in head and neck segments^1^. Each stretch was applied for 25-30 seconds, at high intensity, according to the subjects’ perceptions (score of 8 on a 0-10 scale, for which 0 means no stretching and 10 means a maximum elongation of that muscle).

## Statistical analysis

Sample size was calculated considering PPT as the main outcome, with data from the literature^24^, for a repeated measures design. To detect significant differences of 1 kg/cm^2^ on the PPTs, with standard deviation of 1 kg/cm^2^, a significance level of 0.05, and power of 0.80, the sample size was calculated as at least 10 subjects with TMD. Spanish software was used for calculation (Ene 3.0, Autonoma Barcelona University & Glaxo Smith Kline, Spain). Twelve subjects were included to control a withdrawal rate of 20%.

Shapiro-Wilk and Levene tests were used to respectively evaluate the distribution and homoscedasticity of the numerical variables (pain-free MMO and PPTs). One-factor repeated measures ANOVA was used to compare pain-free MMO and PPTs among evaluations E1, E2, and E3, considering evaluation as within-subject factor. The Tukey test was used for *post-hoc* analysis to locate the difference (E1xE2 or E2xE3). For interpreting those tests, alfa was considered =0.05 (5%). The comparison between E1 and E3 was not performed because it was considered irrelevant to answer the research question.

Friedman test was applied to compare pain and function across evaluations E1, E2, and E3 for numerical ordinal and nonparametric variables (MFIQ and pain scores) considering alfa =0.05 (5%). Moreover, isolated paired comparisons were made (E1xE2 or E2xE3) with Wilcoxon test. Bonferroni correction was applied (α=0.025) to counteract the problem of multiple comparisons (high risk of type I error). Analyses were carried out using the statistical package SigmaPlot (v.11.0).

Finally, effect size (ES) was calculated for normally distributed variables through the relative Cohen’s d coefficient. ES has been defined as “the degree at which the phenomenon is present in the population”. Therefore, the larger the effect size, “the higher the degree at which the phenomenon under study manifests”^14^. ES is the difference in mean scores divided by the pooled standard deviation of the evaluations (evaluations E1 and E2, then evaluations E2 and E3)^4^. Values between 0.2 and 0.49 were interpreted as small effect; those between 0.5 and 0.79 as moderate effect; and values greater than 0.8 as large effect.

## RESULTS

The mandibular function score was different among evaluations (P=0.019). *Post-hoc* analysis revealed significant mandibular function improvement from E2 to E3 (P=0.02) but not from E1 to E2 (P=0.47), as reported in Table 1. Only two subjects presented with worsened function after intervention, while seven showed improvement, and three displayed the same previous MFIQ score.

**Table 1:** Data of Mandibular Function Impairment Questionnaire, self-reported pain, and pain-free Maximum Mouth Opening

	Mean (SD)	ANOVA (P-value)	*post-hoc*	Tukey (P-value)	ES
Maximum Mouth Opening (mm)					
1^st^ Evaluation	31.5 (9.17)	0.002*	1x2	0.88	0.09
2^nd^ Evaluation	32.3 (8.80)				
3^rd^ Evaluation	38.0 (8.82)		2x3	0.009*	0.64
	Median (25%-75%)	Friedman (P-value)	post-hoc	Wilcoxon ♦ (P-value)	
Pain Scale - RDC/TMD					
1^st^ Evaluation	1 (1-3)	0.013*	1x2	0.888	
2^nd^ Evaluation	1 (0-3)				
3^rd^ Evaluation	0 (0-1)		2x3	0.017*	
Mandibular Function – MFIQ					
1^st^ Evaluation	18.5 (11.75 - 24.25)	0.019*	1x2	0.47	
2^nd^ Evaluation	15 (10 - 26.25)				
3^rd^ Evaluation	8.5 (7 - 14.25)		2x3	0.020*	

*: statistically significant values; ES: effect size. ♦ alpha=0.025 (Bonferroni correction); 1x2: Comparison between first and second evaluations; 2x3: Comparison between second and third evaluations

Pain-free MMO also changed with time (P=0.002). *Post-hoc* analysis revealed a significant increase of 5.7 mm (p=0.009) in MMO, from E2 to E3, with moderate effect size (d=0.64). There was no significant difference (P=0.88) and low effect size (d=0.09) between E1 and E2 (Table 1).

In addition to the low level of self-reported pain among subjects (two of them reported no pain at the moment of E1), a significant difference was also found for pain score (P=0.013, Table 1). Self-reported pain decreased in E3 when compared to E2 (P=0.017), with no significant difference between E1 and E2 (P=0.88). Seven subjects showed improvement in this symptom.

Significant differences in PPTs were found in both left temporalis and masseter muscles, and the *post-hoc* analysis showed a significant increase after intervention (P=0.033 and P=0.047, respectively), both with moderate effect size. The comparison between E1 and E2 for PPT data has not shown a statistical difference, and the effect size was low (Table 2).

**Table 2:** Data of Pressure Pain Thresholds

Pressure Pain Thresholds - kg/cm^2^							
Muscle	Evaluations			ANOVA	post-hoc	Tukey	Effect Size
	1^st^mean (SD)	2^nd^mean (SD)	3^rd^mean (SD)	(P-value)		P-value)	
Left masseter	1.25 (0.21)	1.23(0.20)	1.40 (0.27)	0.028*	1x2 2x3	0.896 0.033*	-0.13 0.71
Right masseter	1.41 (0.27)	1.31 (0.28)	1.51 (0.28)	0.105	1x2 2x3	N/A N/A	-0.30 0.65
Left temporalis	1.28 (0.23)	1.32 (0.21)	1.46 (0.20)	0.008*	1x2 2x3	0.714 0.047*	0.19 0.67
Right temporalis	1.64 (0.24)	1.40 (0.24)	1.67 (0.36)	0.003*	1x2 2x3	0.013* 0.060	-1.03 0.91

*: statistically significant values; N/A: not applied. 1x2: comparison between first and second evaluations; 2x3: comparison between second and third evaluations

No significant difference was found on right masseter PPT, but the increasing effect of this measurement was moderate (d=0.65) after treatment. However, right temporalis PPT presented a significant decrease before intervention (P=0.013, d=-1.03) and high effect size after intervention (d=0.91) with no statistical significance (P=0.06).

## DISCUSSION

According to the results, the treatment of the cervical spine based on joint mobilizations, segmental stabilization, and muscle stretching produced statistically significant changes in subjects with TMD. The treatment protocol decreased self-reported pain, increased pain-free MMO, and improved mandibular function. There was also a significant improvement in masticatory muscle sensitivity on the left side.

### Pain and mandibular function

There was a statistically significant improvement in self-reported pain after intervention, with some subjects achieving a median of 0 on the pain scale. Pain was measured by means of the graduate scale included in the RDC/TMD protocol. Even though it is not the most widely used tool for measuring pain, this scale was sensitive to pain variations after intervention. These results agree with the current literature^24^.

The Visual Analogue Scale (VAS) is commonly used in TMD studies. It has been stated that the statistic and clinical success of the treatment requires at least the smallest detectable difference (from 15 to 43 mm, depending on the methodology) and 38% of the initial average pain level^23^. Although all subjects had been diagnosed with TMD, their pain levels were low, causing a ceiling effect. Hence, the clinical relevance for this outcome could not be achieved.

However, pain reduction after intervention was consistent among subjects. Even though the mechanism associated with this result is not fully understood, the stimulation of the inhibitory downward path through the cervical spine has been expected to reduce pain in the trigeminal area. Nevertheless, because signs and symptoms of neck dysfunction were not evaluated in the present study, a relationship between the cervical spine treatment and TMD symptoms cannot be established. Pain reduction is suggested to be associated with increased MMO.

In addition to reduced pain, subjects presented significant improvement in mandibular function after treatment, which may be related to both reduced pain and increased MMO because the MFIQ addresses the major functions of the stomatognathic system (eating different kinds of food, communicating, yawning, and smiling), which require mouth opening and proper performance of this system^11^. Therefore, it is important to consider that the impairment of mandibular function was low among subjects, and the ceiling effect was probably achieved.

Low levels of pain and small impairments of mandibular function are notable characteristics of the college population for which the prevalence of TMD is high^12^ and should not be neglected. Even with low impairment, their difficulty to perform some important activities is common in clinical practice. Furthermore, the investigation of subjects with small severity helps with understanding their symptoms and prevents them from becoming more serious.

### Pain-free MMO

MMO increased an average of 5.7 mm after intervention, corresponding to 17.5% of the initial value with moderated effect size. This improvement agrees with studies that have used manual therapy on the cervical spine in subjects with TMD. A manipulative therapy applied to the upper cervical region of patients with myogenic TMD produced significant improvement of 3.5 mm in MMO immediately after intervention^27^. Moreover, La Touche et al.^24^ (2009) reported an improvement of 4.5 mm after 10 sessions of manual therapy to the cervical spine. Both studies observed clinically important results, although the smallest detectable difference for MMO was reported as 6-9 mm, depending on the evaluation procedure^22^.

A previous study that applied a myofascial release technique on the masseter muscles presented immediate improvements of 4 mm^19^ in MMO. Our protocol induced improvements in jaw opening similar to those observed with local treatment of the masseter muscle. Therefore, an indirect approach focusing on the upper cervical spine may be beneficial to patients with allodynic responses in the orofacial region who find manual application of local interventions extremely painful^27^.

There seems to be a functional integration between jaw and atlanto-occipital movements. Previous studies^16^ ^,^ ^18^ showed that during chewing activities, there are movements on the upper cervical spine related to mouth opening and closing, and they depend on the coordination of the masticatory and cervical muscles. Consequently, the immobilization or alteration of head position can affect mandibular movements^18^.

Moreover, mouth opening is closely related to upper cervical extension^16^. Patients with TMD have shown significant limitations in movements of the upper cervical segment when compared to asymptomatic subjects^15^. Therefore, TMJ alterations can cause neck dysfunction and vice versa. Manual therapy applied to the cervical spine probably contributes to cervical range of motion, facilitating and increasing mouth opening.

Finally, according to Leandri, et al.^26^ (2001), nociceptive impulses from the upper cervical spine cause reflex contractions in masticatory muscles, which can contribute to the development of TMD symptoms. Thus, joint mobilization toward the upper cervical region appears to reduce muscular reflex contractions and to allow muscle relaxation, especially in masseter muscles, and may consequently increase MMO.

Although the improvement on MMO did not reach the clinically meaningful difference^22^, the significant change and the good effect size of the therapy suggest great tendency towards restoration of normal values for MMO, even after few intervention sessions of manual therapy techniques and segmental stabilization applied directly to cervical spine. Limited MMO is one of the main complaints reported by TMD patients because of functional limitations. Therefore, prolonged protocols and other manual therapy techniques to the cervical spine should be considered and investigated for the treatment of TDM patients.

### PPTs

PPTs measured on masseter and temporalis muscles were significantly higher after intervention, particularly on the left side. Moreover, the effect size for both muscles was moderate or large after intervention, but small or nonexistent at the baseline phase. In general, the mean differences were predominantly negative (i.e., there was a reduction in PPTs from the first to second evaluation), although those between the mean values before and after intervention ranged from 0.14 to 0.28 kg/cm^2^, what is lower than the minimal detectable change that varies from 0.45 to 1.13 kg/cm^2^ reported by Walton, et al^31^. Different results were reported by La Touche, et al.^24^ (2009), who found a significant increase (greater than 1.0 kg/cm^2^) in PPTs on both masseter and temporalis muscles after a similar treatment. Another study^27^ that approached TMD patients with upper cervical manipulation verified PPT on the sphenoid bone. The authors reported significant, yet not clinically relevant, results.

PPT of masticatory muscles in TMD patients has been described as lower than those in asymptomatic subjects^30^. Values for masseter muscles are approximately 1.5 kgf/cm^2^ and 2.3 kgf/cm^2^, and for the temporalis muscle, 2.1 kgf/cm^2^ and 3.5 kgf/cm^2^ in TMD and asymptomatic subjects, respectively. Our sample showed a significant increase after treatment with a moderate/large effect from the intervention. However, PPT values for the masseter and temporalis did not reach normal values after treatment. This result suggests some tendency towards improvement that should be better investigated with prolonged protocols, in addition to other manual therapy techniques and control groups.

Finally, PPT of muscles from the left side presented greater improvements than those from the right side. It is important to highlight that the left side was mostly involved in patients presenting joint degenerations or disc displacements associated to myofascial pain. So, the significant increase on PPT of the muscles in the left side after intervention and not in the right one may be related to those associated impairments.

### Study limitations

Sample size estimation was based on PPT data, and statistically significant differences were found when comparing evaluations; however, most differences were lower than the estimated ones and did not show clinical relevance (compared with minimal clinical difference). Therefore, using changes of another outcome to estimate sample size could have provided bigger sample size estimation and different results.

The AB design was supported by the literature^5^ to provide results that could be useful on the clinical decisions for one patient. The design showing the proposed protocol was more effective than time itself because no important changes were observed between first and second evaluations. However, the effectiveness of the protocol should be investigated through a randomized controlled trial, considering either a control or a sham group.

Moreover, 5 weeks of intervention can be considered as associated to short-term results. However, high quality studies investigating manual therapy on subjects with TMD show positive effects with few sessions^8^. We believe that further studies should consider a follow-up performed some weeks after the last evaluation. It can help to understand the perpetuation of the results along time.

Although all subjects were diagnosed with TMD according to the RDC/TMD protocol, they presented with low levels of pain and small impairments of mandibular function. Samples with more severe impairment may have revealed different results. Conversely, the high prevalence of TMD among students and the fact that they are common patients in the daily practice of physiotherapy lead our results to help make the right clinical decision when dealing with this kind of patient.

## CONCLUSIONS

The cervical spine therapy approach using neck joint mobilization, muscle stretching, and segmental stabilization seems to cause significant improvement in pain-free MMO, self-reported pain, and mandibular functionality in subjects with myofascial pain or mixed TMD. Changes showed moderate-to-large effect sizes but small magnitude and no clinical relevance. However, the tendency of the results indicates that further studies should continue investigating the effects of cervical treatment in subjects with TMD. It will bring up stronger evidence about the indirect approach of TMD by physical therapists.
